# FUZZY COMPUTATIONAL MODELS TO EVALUATE THE EFFECTS OF AIR POLLUTION ON CHILDREN

**DOI:** 10.1590/1984-0462/;2018;36;1;00013

**Published:** 2017-11-13

**Authors:** Gleise Silva David, Paloma Maria Silva Rocha Rizol, Luiz Fernando Costa Nascimento

**Affiliations:** a Faculdade de Engenharia, Universidade Estadual Paulista “Júlio de Mesquita Filho”, Guaratinguetá, SP, Brasil.

**Keywords:** Fuzzy logic, Air pollutants, Respiratory tract diseases, Particulate matter, Nitrogen dioxide, Lógica fuzzy, Poluentes do ar, Doenças respiratórias, Material particulado, Dióxido de nitrogênio

## Abstract

**Objective:**

To build a *fuzzy* computational model to estimate the number of hospitalizations of children aged up to 10 years due to respiratory conditions based on pollutants and climatic factors in the city of São José do Rio Preto, Brazil.

**Methods:**

A computational model was constructed using the *fuzzy* logic. The model has 4 inputs, each with 2 membership functions generating 16 rules, and the output with 5 pertinence functions, based on the Mamdani’s method, to estimate the association between the pollutants and the number of hospitalizations. Data from hospitalizations, from 2011-2013, were obtained in DATASUS - and the pollutants Particulate Matter (PM_10_) and Nitrogen Dioxide (NO_2_), wind speed and temperature were obtained by the Environmental Company of São Paulo State (Cetesb).

**Results:**

A total of 1,161 children were hospitalized in the period and the mean of pollutants was 36 and 51 µg/m^3^ - PM_10_ and NO_2_, respectively. The best values of the Pearson correlation (0.34) and accuracy measured by the Receiver Operating Characteristic (ROC) curve (NO_2_ - 96.7% and PM_10_ - 90.4%) were for hospitalizations on the same day of exposure.

**Conclusions:**

The model was effective in predicting the number of hospitalizations of children and could be used as a tool in the hospital management of the studied region.

## RESUMO

**Objetivo:**

Construir um modelo computacional *fuzzy* para estimar o número de internações de crianças até 10 anos por doenças respiratórias, com base nos dados de poluentes e fatores climáticos da cidade de São José do Rio Preto, Brasil.

**Métodos:**

Foi construído modelo computacional utilizando a lógica *fuzzy*. O modelo tem 4 entradas, cada uma com 2 funções de pertinência gerando 16 regras, e a saída com 5 funções de pertinência, baseado no método de Mamdani, para estimar a associação entre os poluentes e o número de internações. Os dados de internações, de 2011-2013, foram obtidos no Departamento de Informática do Sistema de Saúde (DATASUS) e os poluentes material particulado (PM_10_) e dióxido de nitrogênio (NO_2_), a velocidade do vento e a temperatura foram obtidos pela Companhia Ambiental do Estado de São Paulo (Cetesb).

**Resultados:**

Foram internadas 1.161 crianças no período analisado, e a média dos poluentes foi 36 e 51 µg/m^3^ - PM_10_ e NO_2_, respectivamente. Os melhores valores da correlação de Pearson (0,34) e da acurácia avaliada pela curva *Receiver Operating Characteristic* - ROC (NO_2_ - 96,7% e PM_10_ - 90,4%) foram para internações no mesmo dia da exposição.

**Conclusões:**

O modelo mostrou-se eficaz na predição do número de internações de crianças, podendo ser utilizado como ferramenta na gestão hospitalar da região estudada.

## INTRODUCTION

The association between air pollution and respiratory^1^ ^,^ ^2^ ^,^ ^3^ ^,^ ^4^ ^,^ ^5^ ^,^ ^6^ ^,^ ^7^ ^,^ ^8^ and cardiovascular diseases^2^ ^,^ ^9^ is a subject of study, and the global concern about the quality of air has been increasing. In Brazil, the patterns established by the National Environment Council (CONAMA) are followed, in Resolution n. 03/1990,^10^ even if these are above the current patterns adopted in other countries, which updated their legislation. In the State of São Paulo, Decree n. 59,113 is currently valid,^11^and it aims at reaching the standard of the World Health Organization (WHO) using intermediate goals.^12^ ^,^ ^13^

The most studied pollutants that are damaging to health are: particulate matter (PM_10_), nitrogen oxides (NO and NO_2_), sulfur dioxide (SO_2_), ozone (O_3_) and carbon dioxide (CO_2_). In general, the major sources of these pollutants are vehicles, industries, thermoelectric and biomass burn. PM_10_ is composed of solid and liquid particles suspended in the air, lower than or equal to 10 µm, whose fine fraction - PM_2,5_ - reaches the location where gases are exchanged in the lung. NO_2_ is an oxidation agent able to reach the peripheral portions of the lung, and is the precursor of the O_3_ formation.^1^ ^,^ ^4^

From October 2011 to September 2013, there were about 2,7 million hospitalizations caused by respiratory conditions in Brazil; of these, approximately 1.1 million was of children aged up to 10 years - the State of São Paulo alone was responsible for about 200 thousand of them. The expenses with this type of hospitalization in the country, during the same period of this study, was of about 2.5 billion Reais, and approximately one third of this amount was destined to hospitalizations of children aged up to 10 years - of this total, 173 million Reais were spent only in the State of São Paulo.^14^

Statistical techniques, like the Poisson regression, are largely used in studies involving air pollution and health outcomes. In general, these studies demonstrate the association between pollutants and cardiorespiratory conditions, estimating the risk of death or hospitalization.^2^ ^,^ ^3^ The fuzzy approach has been used as an alternative for several areas, such as Medicine. Its great advantage is the facility to handle linguistic terms and inaccurate and uncertain information, besides the low computational cost. Unlike the classic theory, in which each element belongs to a set or not, in the fuzzy logic there is a degree of pertinence, so there may be an element that belongs to a specific group to a higher or lower level.^15^

Like, for instance, the input variable PM_10_ with 24 µg/m^3^. The fuzzy logic allows to attribute to the “acceptable” fuzzy subset a 0.55 level of pertinence, and to the “unacceptable” subset, a 0.45 level of pertinence, resulting in the uncertainties inherent to this record. Indeed, the measurement of the input value of the particulate matter of 23 µg/m^3^ and another of 25 µg/m^3^, which, by the classic logic, would be classified as acceptable and unacceptable, respectively, did not present significant differences. In the fuzzy approach, each element could be compatible with several categories, with different levels of pertinence, making the classification even more realistic.

The objective of this study was to estimate, using a fuzzy model, the number of hospitalizations of children aged up to 10 years for respiratory diseases, based on the data regarding pollutants and environmental factors in the city of São José do Rio Preto, Brazil, strongly affected by the pollution from the burning of sugarcane straw and the roads in the region^12^ ^,^ ^16^ - this city presents with levels of pollution comparable to (and, sometimes, higher than) those found in the Metropolitan Region of São Paulo, as opposed to the paradigm that countryside cities necessarily have better quality of the air.^4^

## METHOD

São José do Rio Preto is in the countryside of the State of São Paulo, to the northwest of the capital (latitude: 20° 48’ S and longitude: 49° 22’ W), with approximately 400 thousand inhabitants and a fleet of about 340 thousand vehicles.^17^ The burning of sugarcane straw is the main cause of air pollution in this region - there are about 130 thousand hectares/year -, and the black smoke resulting from the burning of fuel by diesel motors is the second biggest cause. The administrative region of São José do Rio Preto has an agro-industrial profile based mainly on the production of sugar and alcohol, from the sugarcane, and on the manufacture of furniture.^16^

A computational fuzzy model was developed to assess the number of hospitalizations caused by respiratory disease in children aged up to 10 years, according to the concentrations of PM_10_ and nitrogen dioxide (NO_2_), and the values of air temperature and wind speed, obtained from the Environmental Company of São Paulo State (Cetesb), in São José do Rio Preto. The database of the number of hospitalizations caused by respiratory diseases in children was obtained from the website of the Department of Informatics in the Unified Health System (DATASUS)^14^ for the diseases in chapter X of the International statistical classification of diseases and related health problems - 10th revision (J00 - J99), for the city of São José do Rio Preto, from October 1^st^, 2011, to September 30, 2013.

**Figure 1:**
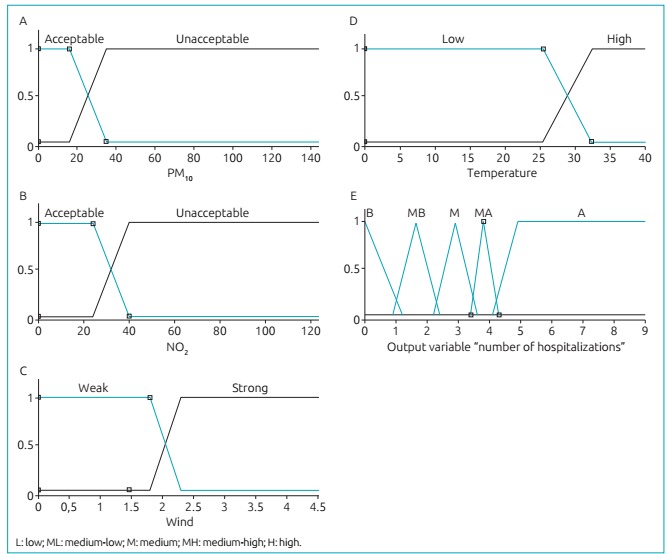
Input variables in the system of fuzzy inference with the level of pertinence in axis y - (A) particulate matter (µg/m³), (B) nitrogen dioxide (µg/m³), (C) wind speed (m/s²) and (D) air temperature and output (E) number of hospitalizations for respiratory diseases in children, São José do Rio Preto, Brazil 2011-2013.

The system inputs are presented in Figure 1 (A-D): PM_10_, NO_2_, wind speed and temperature, respectively; and, in Figure 1E, the output represented by the number of hospitalizations caused by respiratory diseases in children living in São José do Rio Preto. Table 1 shows the maximum and minimum values, as well as the standard deviation of the data used.

**Table 1:** Values of means, standard deviation, minimum and maximum of particulate matter, nitrogen dioxide, temperature and wind speed, and real number of hospitalizations (Real) and estimated by model (Model), São José do Rio Preto, Brazil, 2011-2013.

	Mean	Standard deviation	Minimum	Maximum
PM_10_ (µg/m^3^)	36.57	22.12	6.00	144.00
NO_2_ (µg/m^3^)	51.35	23.46	7.00	124.00
Temperature (ºC)	30.38	3.66	11.50	39.80
Wind (m/s^2^)	2.26	0.53	1.10	4.10
Number of hospitalizations (Real)	1.59	1.55	0.00	9.00
Number of hospitalizations (Model)	3.24	1.81	0.37	6.77

PM_10_: particulate matter; NO_2_: nitrogen dioxide.

The four input variables were “fuzzified” with two trapezoidal pertinence functions each, and the output has five pertinence functions (four triangular and one trapezoidal), according to the expert. Therefore, 16 rules were defined (2x2x2x2), considering the effects of the pollutants and the climactic variables in respiratory diseases in children. The rules relating the inputs and outputs are shown in Table 2.

**Table 2:** Base of rules in the fuzzy model inserted in Matlab. São José do Rio Preto, Brazil, 2011- 2013.

1. If (PM_10_ is Acceptable) and (Temperature is High) and (NO_2_ is Acceptable) and (Wind is Strong) then (Number of hospitalizations is L) (1)
2. If (PM_10_ is Acceptable) and (Temperature is High) and (NO_2_ is Acceptable) and (Wind is Weak) then (Number of hospitalizations is L) (1)
3. If (PM_10_ is Acceptable) and (Temperature is Low) and (NO_2_ is Acceptable) and (Wind is Strong) then (Number of hospitalizations is L) (1)
4. If (PM_10_ is Acceptable) and (Temperature is Low) and (NO_2_ is Acceptable) and (Wind is Weak) then (Number of hospitalizations is L) (1)
5. If (PM_10_ is Acceptable) and (Temperature is High) and (NO_2_ is Unacceptable) and (Wind is Strong) then (Number of hospitalizations is ML) (1)
6. If (PM_10_ is Acceptable) and (Temperature is High) and (NO_2_ is Unacceptable) and (Wind is Weak) then (Number of hospitalizations is M) (1)
7. If (PM_10_ is Acceptable) and (Temperature is Low) and (NO_2_ is Unacceptable) and (Wind is Strong) then (Number of hospitalizations is M) (1)
8. If (PM_10_ is Acceptable) and (Temperature is Low) and (NO_2_ is Unacceptable) and (Wind is Weak) then (Number of hospitalizations is M) (1)
9. If (PM_10_ is Unacceptable) and (Temperature is High) and (NO_2_ is Acceptable) and (Wind is Strong) then (Number of hospitalizations is ML) (1)
10. If (PM_10_ is Unacceptable) and (Temperature is High) and (NO_2_ is Acceptable) and (Wind is Weak) then (Number of hospitalizations is ML) (1)
11. If (PM_10_ is Unacceptable) and (Temperature is Low) and (NO_2_ is Acceptable) and (Wind is Strong) then (Number of hospitalizations is M) (1)
12. If (PM_10_ is Unacceptable) and (Temperature is Low) and (NO_2_ is Acceptable) and (Wind is Weak) then (Number of hospitalizations is M) (1)
13. If (PM_10_ is Unacceptable) and (Temperature is High) and (NO_2_ Unacceptable) and (Wind is Strong) then (Number of hospitalizations is M) (1)
14. If (PM_10_ is Unacceptable) and (Temperature is High) and (NO_2_ Unacceptable) and (Wind is Weak) then (Number of hospitalizations is MH) (1)
15. If (PM_10_ is Unacceptable) and (Temperature is Low) and (NO_2_ Unacceptable) and (Wind is Strong) then (Number of hospitalizations is MH) (1)
16. If (PM_10_ is Unacceptable) and (Temperature is Low) and (NO_2_ Unacceptable) and (Wind is Weak) then (Number of hospitalizations is H) (1)

L: low; medium-low: ML; M: medium; MA: medium-high; H: high.

The input variables are “fuzzified” by pertinence levels. Then, the Mamdani’s inference process is conducted, also known as maximum and minimum. Finally, the centroid “defuzzification” method is applied to obtain the output (number of hospitalizations).^15^

For the validation of the model, the Pearson correlation was performed between the real data and the fuzzy model, with 0 to 3-day lag - that is, from the day of exposure to the third day - after the inhalation of pollutants. The ROC curves of the pollutants were also assessed, with a cutoff point of up to 2 hospitalizations, in which values referring to the accuracy of the model with 5% significance level were obtained.

## RESULTS

In the period assessed, 1,161 hospitalizations of children caused by respiratory diseases were reported in the city of São José do Rio Preto, generating expenses of about 1.7 million reais, that is, approximately 1% of the expenses of the State of São Paulo were addressed to this type of hospitalization.^14^

**Figure 2:**
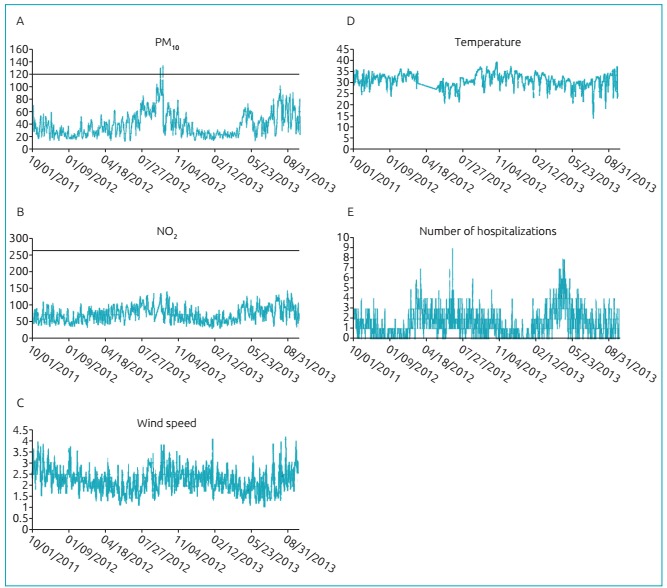
Temporal distribution of the values of variables - (A) particulate matter (µg/m³), (B) nitrogen dioxide (µg/m³), (C) wind speed (m/s^2^) and (D) wind temperature (ºC) - and (E) number of hospitalizations of children, São José do Rio Preto, Brazil, 2011-2013.

Figure 2 shows the distribution of values for PM_10_, NO_2_, temperature, wind speed and number of hospitalizations. It is possible to observe some seasonality in the concentrations of pollutants. In the period known as the burn of biomass and the lack of rains (between July and September), there is increase in pollutants, usually attributed to this burning practice to facilitate the manual sugarcane harvesting. Therefore, the increasing amount of pollutants leads to the increasing number of hospitalizations; temperature and wind show an inverse relationship: its increase reduces the number of hospitalizations.

The Pearson correlation between the real number of hospitalizations and the model presented significant results for the 0 to 3-day lag, and the best correlation was r=0.34 for the 0-day lag, followed by the r=0.29 correlation for the 1-day lag, r=0.29 for the 2-day laf, and r= 0.27 for the 3-day lag (p-value<0.05). 

The area values under the ROC curve for the 0 to 3-day lags are presented in Table 3, observing that the best performance was that of the 0 lag of 96.7% (95%CI 95.4-98.0) for NO_2_ and 90.4% (95%CI 88.1-92.6) for PM_10_.

**Table 3:** ROC curve values and respective 95% confidence intervals for 0 to 3-day lags of the particulate matter pollutants and nitrogen dioxide, São José do Rio Preto, Brazil, 2011-2013.

	Lag 0	Lag 1	Lag 2	Lag 3
PM_10_	0.904 (0.881 - 0.926)	0.775 (0.739 - 0.811)	0.730 (0.691 - 0.769)	0.709 (0.669 - 0.749)
NO_2_	0.967 (0.954 - 0.980)	0.803 (0.769 - 0.838)	0.716 (0.675 - 0.756)	0.684 (0.641 - 0.726)

PM_10_: particulate matter; N_O2_: nitrogen dioxide.

## DISCUSSION

This study showed the feasibility of the fuzzy logic application for the prediction of the number of hospitalizations of children for respiratory diseases, based on the concentrations of pollutants and on the values of temperature and wind speed.

In the literature, it is possible to find previous studies that approached the effects of air pollution on the hospitalizations caused by respiratory diseases in children^2^ ^,^ ^3^ ^,^ ^7^ using Poisson regression methods for temporal series. There are also studies that used the fuzzy approach to estimate the period of hospitalization caused by respiratory diseases^5^ and heart conditions^9^, besides risk of neonatal death.^18^ ^,^ ^19^ This study showed the number of hospitalizations of children caused by pollutants according to 0 to 3-day lags. The fuzzy model built relates the exposure to air pollutants, temperature and wind and the number of hospitalizations for respiratory diseases in children, showing good performance in the prediction of the number of hospitalizations with lags of up to 3 days.

The mean of concentrations of pollutants in São José do Rio Preto is comparable to the mean in the city of São Paulo. In the analyzed period, the mean concentration of pollutants in the city of São Paulo was 33 µg/m^3^ for PM_10_ and 75 µg/m^3^ for NO_2._ In São José do Rio Preto it was 36 µg/m^3^ for PM_10_ and 51 µg/m³ for NO_2_. In this period, the concentration of PM_10_ was considered moderate (>50 µg/m^3^) in 164 days (22.4% of the studied days), poor (>100 µg/m^3^) on 7 days (0.1% of the studied days), and crossed the limit for 2 dayss (>120 µg/m^3^). Regarding the mean concentration of the pollutant NO_2_, it remained within the values accepted by the current law in the entire studied period.^13^ ^,^ ^20^

The sensitivity of the model built for the pollutants PM_10_ and NO_2_, assessed by the ROC curve, presented good value. Accuracy was significant for the 0-day lag with NO_2_, with an area under the 96.7% curve, sensitivity of about 92.0%, and approximate specificity of 90.0%.

It is important to consider that the number of hospitalizations obtained from data coming from Datasus^14^ does not include hospitalizations by private insurance plans and other health operators, outpatient treatments and errors in address (which is common in central cities, in which individuals claim to live in different city). However, these data are used in several studies, and are sufficient for the model proposed.

One limitation of this study is that the concentration of pollutants measured may not represent the entire territory of São José do Rio Preto. For this study, however, the concentration was considered homogeneous. Other factors, such as the predisposition to respiratory diseases and pollution in closed environments were not considered.

On the other hand, this study presents a low-cost and feasible model, which can be used in any town that provides data (pollutants and environmental factors) using applications for tablets or smartphones, for example. It can also be used to help the public health management, providing health teams and public policies managers with an estimation of the expected number of hospitalizations.
